# RETRACTION: Protective Effect of Sundarban Honey against Acetaminophen‐Induced Acute Hepatonephrotoxicity in Rats

**DOI:** 10.1155/ecam/9863412

**Published:** 2026-07-20

**Authors:** Evidence-Based Complementary and Alternative Medicine

RETRACTION: R. Afroz, E. M. Tanvir, M. F. Hossain, et al., “Protective Effect of Sundarban Honey against Acetaminophen‐Induced Acute Hepatonephrotoxicity in Rats,” *Evidence-Based Complementary and Alternative Medicine*, 2014, 143782, https://doi.org/10.1155/2014/143782.

The above article, published online on 30 October 2014 in Wiley Online Library (https://wileyonlinelibrary.com), has been retracted by John Wiley & Sons Ltd. The retraction has been agreed due to multiple concerns identified with Figures 2 and 3, raised on PubPeer [1]. Specifically:

• In Figure 2, panels a and b appear to be identical
• In Figure 3, panels a and b appear to be identical
• In Figure 3, panels A and B appear to be identical to panel D in Figure 4 of [2], by some of the same authors
• In Figure 3, panel D is similar to panels E and F in Figure 4 of [2], after rotation

Following assessment of the concerns, and in the absence of the original images from the authors, the results and conclusions can no longer be considered reliable. The article is therefore retracted.

The authors agree to the retraction.
